# Effects of aging on corneal parameters measured with Pentacam in healthy subjects

**DOI:** 10.1038/s41598-019-39234-x

**Published:** 2019-03-04

**Authors:** Géza Vitályos, Bence Lajos Kolozsvári, Gábor Németh, Gergely Losonczy, Ziad Hassan, Dorottya Pásztor, Mariann Fodor

**Affiliations:** 1 Department of Pediatric Dentistry and Orthodontics, Faculty of Dentistry, University of Debrecen, Nagyerdei krt. 98, 4012 Debrecen, Hungary; 2 Department of Ophthalmology, Faculty of Medicine, University of Debrecen, Nagyerdei krt. 98, 4012 Debrecen, Hungary; 3 Department of Ophthalmology, Borsod-Abaúj-Zemplén County Hospital and University Teaching Hospital, Szentpéteri gate 72-76, 3526 Miskolc, Hungary; 4 Department of Ophthalmology, Zuyderland Hospital, Dr. H. van der Hoffplein 1, 6162 BG Geleen, The Netherlands; 5 Orbident Refractive Surgery and Medical Center, Nagyerdei krt. 98, 4012 Debrecen, Hungary

## Abstract

Our purpose was to prospectively analyze the age-related changes of corneal Scheimpflug parameters in healthy subjects. Thirty-five eyes of 35 volunteers (age 14–67 years) were investigated with an average interval of 3.6 years. Changes of corneal parameters (flattest keratometric reading at anterior (K1F) and posterior surface (K1B), steepest keratometric reading at anterior (K2F) and posterior surface, anterior astigmatism, posterior astigmatism (AstigB), flat axis of anterior and posterior astigmatism (AxisB), thinnest pachymetric value (PachyMin), corneal volume (CV10-mm)) were analyzed. K1F and K2F decreased significantly during observation and showed stronger decrease in younger than in older individuals. Higher values proved to be more stable. K1B decreased significantly and the degree of decrease was dependent on its baseline value and age: in young subjects low values increased, high values decreased. AstigB decreased significantly and showed a baseline-dependent significant increase from lower and a significant decrease from higher initial values. Over time, the mean AxisB shifted significantly. PachyMin and CV decreased significantly with age, especially from higher baseline values in younger subjects. The results of this longitudinal study suggest that both corneal surfaces change significantly with age. We demonstrate for the first time that age and baseline values influence age-related changes of corneal parameters.

## Introduction

Aging is a physiological process and occasionally it is hard to differentiate between time dependent biological changes and damages from environmental insults^1^. Age-related changes occur in all structures of the eye, with various consequences^2^. Age has been identified as an important individual variable affecting the outcome after keratorefractive surgery^3^.

Corneal aging generates structural and functional changes including steepening of keratometry indices, and a rotation of the axis of astigmatism resulting in a shift from with-the-rule to against-the-rule astigmatism^1,4–11^. Alterations of higher-order aberrations of the cornea are also well known^9^. The level of astigmatism decreased significantly with age both of the anterior and posterior corneal surfaces in a study by Nemeth *et al*.^9^, although age as a primary factor was not analyzed in this report.

To differentiate between physiological and pathological changes of the aging cornea, it is indispensable to know what the physiological changes are. There are clinical situations where it is important to distinguish between pathological and normal age-related changes. The assessment of the progression of ectatic corneal disorders in young patients determines the optimal treatment and the glaucoma management in the elderly can only be personalized if we take account the changes of corneal thickness. A complete and precise evaluation of the cornea must take keratometric, astigmatic vectorial as well as pachymetric characteristics into account.

Age-dependent change of corneal thickness has been studied extensively. Corneal thickness decreases throughout infancy; during babyhood (around 3 years of age) it reaches adult thickness and, from that point on, central corneal thickness (CCT) appears to be stable over time^12^. However, age-related change in corneal thickness appears to be rather controversial in other studies^13–18^. CCT has been extensively investigated in glaucoma development in the elderly, and it is known to directly affect the strategy of glaucoma management^13,14,19^. Corneal ectatic disorders are characterized by progressive deformation of the corneal architecture and corneal thinning. Therefore, corneal thinning was extensively studied in young keratoconus patients^20,21^. Scheimpflug-based tomography provides corneal thickness map and curvature maps of both the anterior and the posterior surfaces which improves the sensitivity and specificity of keratoconus detection^11,21,22^.

Several studies examined normal age-related changes of corneal parameters (i.e. CCT, astigmatism, higher-order aberrations), most of what were cross sectional and not longitudinal in nature^4,7–10,13,16,19,23,24^. In the present study we determine age-related changes of corneal parameters using a Pentacam device in a healthy cohort aged between 14 and 67 years at baseline. To the best of our knowledge, this is the first longitudinal study evaluating age-related changes of corneal parameters measured with the Pentacam HR device covering a five-decade range of baseline age in healthy participants.

## Patients and Methods

### Subjects and clinical examinations

Our study comprised 35 healthy participants of European descent with 20/20 Snellen equivalent distance visual acuity with low refraction error (lower than 1.5 diopters [D]) but without other ophthalmological disorders. Following the tenets of the Declaration of Helsinki, written informed consent was signed by all participants and/or their parent and/or legal guardian for study participation prior to enrollment. The study protocol was approved by the Regional and Institutional Research Ethics Committee of the University of Debrecen (DEOEC-RKEB/IKEB 3313-2011). Exclusion criteria were: refraction error more than 1.5 D (including pathologic myopia and hyperopia), active inflammatory or infective systemic or ocular disease, current treatment with systemic or local drugs, use of eyedrops, contact lens wear, previous ocular surgery, abnormality in the lens or retina on biomicroscopic examination, precedent chemical injury or delayed epithelial healing, age less than 14 years, and pregnancy or lactation. Prior to a standard ophthalmological investigation, three images were captured of one randomly selected eye of each patient with high-resolution Pentacam (Pentacam HR, Oculus Optikgeräte GmbH, Wetzlar, Germany, software version 1.17r139) using a 12-mm wide Scheimpflug imaging technique. The device was set to a 25 images/second mode and images were taken in auto mode at perfect eye-set. In case of image distortion (e.g. blinking) or lack of data, the snapshot was repeated. The Pentacam evaluates five different parameters. The quality of the measurement is evaluated for each parameter by the equipment. A measurement is only considered reliable, if the quality of all the five parameters is approved by the equipment by giving an OK signal to the examiner. Data from all three images per session were averaged into session-level values.

All participants underwent repeated ophthalmological examination during a follow-up period of 3.6 years on average. At the baseline and follow-up visit the following parameters were recorded for each eye: examination date, Snellen visual acuity, spherical equivalent. The following data were exported from Pentacam to Microsoft Excel (Microsoft Corp, Redmond, Washington): Holladay equivalent keratometry values in the flat (K1) and steep (K2) meridian of the front and the back surface, maximal keratometry values of the front surface (Kmax), corneal astigmatism of the front and the back surface (Astig F and Astig B, respectively), corneal thickness at the thinnest point of the cornea (Pachy Min), the volume of the cornea in a diameter of 10 mm centered on the anterior corneal apex (C Vol D 10 mm), index of surface variation (ISV), index of vertical asymmetry (IVA), index of height asymmetry (IHA) and index of height decentration (IHD). The change in Pentacam parameters were analyzed always in comparison with the baseline values.

CCT is known to decrease throughout the day, with highest values found in the morning^25^. Therefore, all examinations in this study were performed between 8 and 10 am to correct for daily corneal thickness changes.

### Statistical methods

Outcome parameters reported by the Pentacam system were mostly treated as untransformed continuous variables on their natural scales and units. Exceptions included corneal axis angles, which were consolidated into the range 0° to 90° to derive a laterality-independent measure of location within the range running from horizontal to vertical; and posterior corneal astigmatism values, which were sign reversed. Jackson’s cross cylinder power vector components (J_0_ and J_45_) were calculated for the anterior corneal surface (i.e., the simulated keratometric data) with the method described by Thibos *et al*.^5^. Unadjusted comparisons of follow-up vs baseline readings were based on paired t-tests if normality assumptions were satisfied or Wilcoxon’s matched-pairs signed-ranks tests otherwise. Male subjects were compared to females in terms of age using Wilcoxon’s rank-sum test. Multilevel mixed-effects linear regression was used for adjusted estimation of changes in outcome parameters through follow-up time. Adjustment variables included baseline readings of the outcome parameter, baseline age, and interaction terms between age and follow-up time, and between baseline reading and follow-up time (sex proved unnecessary to adjust for). Findings were expressed as estimated annual changes with 95% confidence intervals either as a function of baseline variable(s) (in presence of a significant interaction) or as a single overall estimate (in absence of significant interactions), and visualized using scatter plots of baseline reading of outcome parameter vs. age at baseline, with symbology indicating direction, significance, and magnitude of change over time. The statistical package applied was Stata version 11. The significance criterion was set at α < 0.05.

### Ethical approval

The study protocol had been approved by the ethical board of University of Debrecen.

### Statement of human and animal rights

The study protocol and execution complied with the Declaration of Helsinki.

### Informed consent

All patients provided informed consent.

## Results

Our study included thirty-five randomly selected eyes of 35 healthy volunteers of European descent (22 women and 13 men; 19 right and 16 left eyes). Participants were predominantly female (63%). There was no statistically significant difference regarding age between genders (p = 0.348). The mean age at the first visit was 31.8 years (SD: 12.4, range: 14.2–66.9 years). The average time interval between visits was 3.6 years (SD: 0.92, range: 2.1–5.7 years). The mean age at the control visit was 35.4 years (SD: 12.3, range: 16.4 to 69.2 years).

Detailed keratometric results of the anterior and posterior corneal surfaces and the differences between the two visits are displayed in Table 1. Both keratometric values (K1 and K2) on the anterior corneal surface decreased significantly during observation (p = 0.002 and 0.048, respectively), with no accompanying changes to K max. No significant changes in K1 and K2 were observed with unadjusted analysis on the posterior surface. The astigmatism parameter only changed significantly with aging on the posterior surface of the cornea (p = 0.022). The mean deviation from horizontal of the flat axis angle on the posterior surface (K1 B) was 8.5° (SD = 7.3°, range: 0.9–35.4°) at baseline and 10.2° (SD = 7.3°, range: 1.6–33.3°) at the end of follow-up. The difference was significant (p = 0.027). There was no significant difference between baseline and follow-up for the flat axis angle on the anterior corneal surface (K1 F) (p = 0.602). Regarding power vectors of the anterior surface, Jackson’s cross cylinder power vector component at 45° demonstrated weak changes with advancing age (p = 0.047).

**Table 1:** Keratometric and vector parameters in healthy subjects (N = 35) measured with Pentacam HR at baseline and after an average 3.6 years of follow-up.

	Baseline mean (SD)	Follow-up mean (SD)	p
K1 F (D)	43.53 (1.28)	43.42 (1.29)	**0.002** ^*^
K2 F (D)	44.39 (1.48)	44.33 (1.49)	**0.048** ^*^
K max (D)	44.91 (1.58)	44.91 (1.65)	0.944
K1 B (D)	−6.20 (0.22)	−6.21 (0.21)	0.741
K2 B (D)	−6.52 (0.28)	−6.50 (0.26)	0.061
Astigmatism F (D)	0.86 (0.53)	0.90 (0.55)	0.284
Astigmatism B (D)	−0.32 (0.13)	−0.29 (0.11)	**0.022** ^*^
J0	0.36 (0.30)	0.37 (0.33)	0.821
J45	0.024 (0.18)	0.048 (0.18)	**0.047** ^*^

K1 = flat-axis keratometric value in diopters (D) on anterior (F) and posterior (B) corneal surface; K2 = steep-axis keratometric value in diopters (D) on anterior (F) and posterior (B) corneal surface; Kmax = maximum simulated keratometry reading in diopters (D); J0 = Jackson’s cross cylinder power vector component at 0 degrees for the anterior corneal surface; J45 = Jackson’s cross cylinder power vector component at 45 degrees for the anterior corneal surface; SD = standard deviation; p = p-value of paired test for follow-up vs baseline.

*Significant difference.

The examination of pachymetric features of the cornea revealed that corneal thickness at the thinnest point of the cornea (Pachy min) decreased significantly with age (p < 0.001) (Table 2). In line with this, corneal volume in the central 10 mm also decreased significantly with age (p < 0.001). During follow-up, the Pentacam indices IHA and IHD showed significant increase (p ≤ 0.001), while ISV and IVA did not change (Table 2).

**Table 2:** Pachymetric and volumetric corneal parameters and indices in healthy subjects (N = 35) measured with Pentacam HR at baseline and after an average 3.6 years of follow-up.

	Baseline mean (SD)	Follow-up mean (SD)	p
Pachy min (μm)	548 (31.5)	534 (29.6)	**<0**.001^*^
C Vol D 10 mm	61.1 (3.7)	60.0 (3.4)	**<0**.001^*^
IHA	4.5 (3.7)	6.4 (4.5)	**0**.001^*^
IHD	0.008 (0.005)	0.012 (0.007)	**<0**.001^*^
ISV	17.0 (5.7)	16.7 (6.2)	0.123
IVA	0.123 (0.057)	0.128 (0.059)	0.567

Pachy min = corneal thickness at the thinnest point of the cornea (μm); C Vol D 10 mm = volume of the cornea in a diameter of 10 mm, centered on the anterior corneal apex; IHA = index of height asymmetry; IHD = index of height decentration; ISV = index of surface variation; IVA = index of vertical asymmetry; SD = standard deviation; p = p-value of paired test for follow-up vs baseline.

^*^Significant difference.

**Figure 1:**
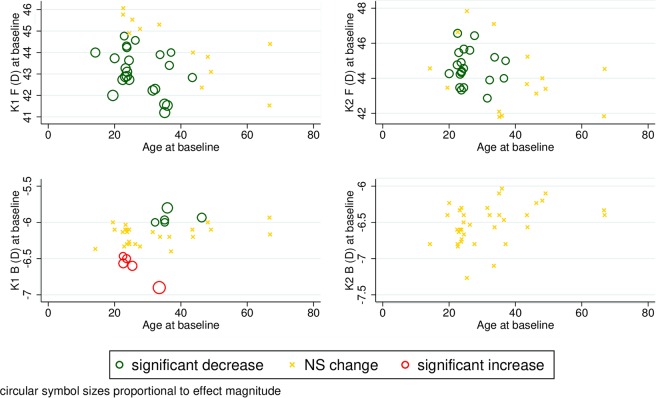
Scatter plots of anterior and posterior K1 and K2 parameters of normal healthy corneas against age with model-predicted tendencies of change over time. K1 = flat-axis keratometric value in diopters (D) on anterior (F) and posterior (B) corneal surface; K2 = steep-axis keratometric value in diopters (D) on anterior (F) and posterior (B) corneal surface; NS = non-significant.

Taking baseline age into account, the front keratometric values (K1 and K2) decreased more substantially in younger than in older individuals, as shown in Fig. 1. Although unadjusted analysis shows no significant changes in K1 and K2 on the posterior surface, adjusted statistical modeling revealed that the keratometric reading on the posterior corneal surface in flat axis (K1B) changes in a heterogeneous fashion across baseline value and, to some extent, age. Low-range keratometric values, which are observed in younger subjects, increase significantly with time, while those in the high range do the opposite (Fig. 1).

**Figure 2:**
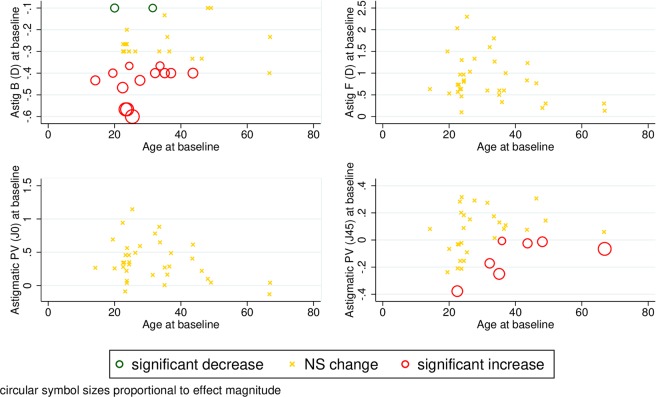
Scatter plots of anterior and posterior astigmatism parameters and the power vectors J0 and J45 of normal healthy corneas against age with model-predicted tendencies of change over time. Astig F (D) = corneal astigmatism of the front surface in diopters, Astig B (D) = corneal astigmatism of the back surface in diopters, J0 = Jackson’s cross cylinder power vector component at 0 degrees for the anterior corneal surface; J45 = Jackson’s cross cylinder power vector component at 45 degrees for the anterior corneal surface; NS = non-significant.

Neither the keratometric astigmatism parameter on the anterior surface of the cornea nor the J0 vector changed significantly with time. Adjusted analysis also shows that these two readings are stable across follow-up throughout the entire range of baseline readings and ages (Fig. 2). The borderline significant change detected in Jackson’s cross cylinder power vector component at 45° (unadjusted p = 0.047) is explained by a significant increase in older subjects with low to mid-range initial J45 values; values in younger subjects and in those with mid to high-range baseline readings did not change with advancing age (Fig. 2). Investigation into age-related progression of posterior corneal surface astigmatism revealed baseline dependent tendencies: if the baseline value of the astigmatism parameter was low, it tended to significantly increase, and if it was high, to significantly decrease during follow-up; this was true across the whole range of baseline age in our sample (Fig. 2).

**Figure 3:**
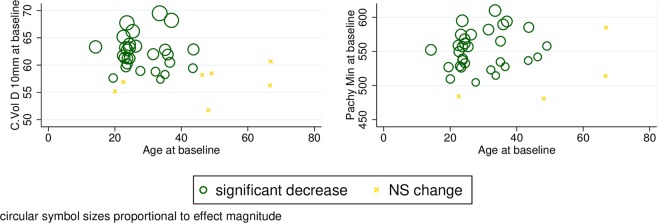
Scatter plots of the C Vol D 10 mm and Pachy min parameters of normal healthy corneas against age with model-predicted tendencies of change over time. C Vol D 10 mm = the volume of the central 10 mm diameter of the cornea; Pachy min = corneal thickness at the thinnest point of the cornea (μm); NS = non-significant.

Adjusted analysis shows that both the volume of the central 10 mm diameter of the cornea and the Pachy min parameter decrease significantly, especially from higher baseline measured values, with some suggestion of dependence on baseline age. Readings that are low at baseline, and also those registered in older subjects, seem to have a limited capacity to change (Fig. 3).

Based on our analysis, the estimated annual change of Pentacam parameters of a normal, healthy eye are demonstrated in Table 3. It is found that, on the anterior surface of the cornea, K1 and K2 decrease significantly (although maintaining a stable Kmax); on the posterior surface, however, only high-range values of the flat keratometry parameter (K1) decrease significantly over time (p = 0.019). Front astigmatism seems to remain stable with aging, while higher initial absolute values of posterior astigmatism tend to reduce. Anterior axes of astigmatism do not seem to vary over time; initially horizontal posterior axes shift significantly towards the vertical, and those in the vertical or oblique angle at baseline do so towards the horizontal position. While J0 seems to be stable, J45 significantly increases from a low baseline (p = 0.012). The volume of the central 10 mm diameter of the cornea and the Pachy min parameters decrease stronger in thicker corneas.

**Table 3:** Estimated annual changes from baseline in Pentacam measurement parameters based on a sample of healthy subjects (N = 35).

outcome parameter	baseline of outcome	estimated annual change	95% CI	p
K1 F (D)	sample average	−0.027	−0.043; −0.012	**0**.001^*^
K2 F (D)	sample average	−0.022	−0.035; −0.009	**0**.001^*^
K1 B (D)	−6.4	0.005	−0.001; 0.011	0.10
	−6.1	−0.003	−0.008; 0.002	0.20
	−6.0	−0.008	−0.014; −0.002	**0**.019^*^
K2 B (D)	sample average	0.003	−0.002; 0.008	0.26
K max (D)	sample average	−0.002	−0.025; 0.021	0.87
Astigmatism F (D)	sample average	0.005	−0.013; 0.022	0.61
Axis F (flat)(°)	sample average	0.282	−0.47; 1.034	0.46
J0	sample average	−0.001	−0.010; 0.008	0.87
J45	−0.15	0.013	0.003; 0.024	**0**.012^*^
	0.01	0.006	−0.001; 0.013	0.10
	0.18	−0.001	−0.010; 0.007	0.76
Astigmatism B (D)	−0.40	0.012	0.005; 0.018	**0**.001^*^
	−0.30	0.004	−0.002; 0.009	0.19
	−0.23	−0.002	−0.008; 0.005	0.61
Axis B (flat) (°)	0	1.342	0.507; 2.177	**0**.0016^*^
	45	−3.6	−6.225; −0.975	**0**.0072^*^
	90	−8.542	−14.340; −2.743	**0**.0039^*^
Pachy min	524	−2.480	−3.541; −1.419	<**0**.001^*^
	550	−3.279	−4.182; −2.377	<**0**.001^*^
	581	−4.228	−5.538; −2.918	<**0**.001^*^
C Vol D 10 mm	58	−0.168	−0.264; −0.072	**0**.001^*^
	61	−0.268	−0.347; −0.189	<**0**.001^*^
	63	−0.341	−0.438; −0.244	<**0**.001^*^
IHA	sample average	0.569	0.298; 0.840	<**0**.001^*^
IHD	sample average; age 22 sample average; age 35 sample average; age 50	0.001	0.001; 0.002	<**0**.001^*^
		0.001	0.001; 0.001	<**0**.001^*^
		0.000	−0.000; 0.001	0.41
ISV	sample average	−0.079	−0.257; 0.098	0.38
IVA	sample average	0.002	−0.001; 0.004	0.26

K1 = flat-axis keratometric value in diopters (D) on anterior (F) and posterior (B) corneal surface; K2 = steep-axis keratometric value in diopters (D) on anterior (F) and posterior (B) corneal surface; K max = maximal keratometry values of the front surface; J0 = Jackson’s cross cylinder power vector component at 0 degrees; J45 = Jackson’s cross cylinder power vector component at 45 degrees; Pachy min = corneal thickness at the thinnest point of the cornea (μm); C Vol D 10 mm = the volume of the central 10 mm diameter of the cornea; IHA = index of height asymmetry; IHD = index of height decentration; ISV = index of surface variation; IVA = index of vertical asymmetry; 95% CI = 95% confidence interval; p = p-value of estimated change; *Significant change.

## Discussion

A wide range of changes occur in the aging cornea, some of which are clinically relevant for planning surgical procedures such as correcting astigmatism and myopia, for glaucoma management, and also for the management of corneal ectatic disorders. Reports on age-related changes of corneal thickness in healthy eyes are somewhat contradictory. Central corneal thickness seemed to be stable (mean change of CCT was −1.9 ± 14 μm) over 1.5 years in children^12^, while other studies reported no significant association between corneal thickness and age^17,26^. On the other hand, Rieth *et al*. detected significantly increased corneal thickness in the elderly in a cross sectional study^16^. In line with our observations, vast majority of previous studies report a statistically significant inverse relationship between age and corneal thickness^13–15,18,23,24^. In the present work we detected a 14-μm average decrease at the thinnest point of the cornea representing the strongest decrease reported in longitudinal studies so far (5 to 14 μm across 8.2 years, 3.5 μm across 3.8 years, 2.6 μm across 5 years reported by Weizer, Brandt and Hashemi *et al*., respectively)^14,15,18^. The mean ages of the longitudinal study samples were 50 to 60 years at the baseline visit^14,15,18^, which may explain why our younger patient population (mean age 31.8 years) showed more pronounced corneal thinning. This observation indicates that corneal tissue degradation is more pronounced in younger ages. There is only one study that analyzed thinnest point thickness change with a Pentacam device^18^. At the baseline in this study individuals were between 41 and 64 years old (average 49.9 years) and had a mean corneal thickness of 524.9 μm (SD 32), which is 13 μm thinner than in our younger study group of 14 to 67-year-old subjects (average 31.8 years). The difference in corneal thickness between these two different age groups confirms our observation on the age dependent corneal thinning found in our longitudinal study.

Little attention was paid to corneal volume in earlier studies, although it may sensitively follow topographical and pachymetric changes of the cornea and therefore is a sensitive indicator of corneal health^24,27^. Recently, the change in this parameter was assessed during accommodation, in connection with phacoemulsification, refractive surgery, keratoconus and anatomical deformations^28–31^. No longitudinal studies determined age-related changes in corneal volume in a healthy cohort so far. In the present study we found an age-dependent decrease in corneal volume of the central 10 mm. Interestingly the change was more pronounced in case of higher baseline values and younger individuals. This change is very similar to that observed with corneal thickness however their reason is still largely unknown^24^. The thinner cornea in line with the higher degree of rigidity in older aged patients might be a hint of physiologically cross linking process in elderly individuals. Investigation of the age-related, long-term changes of corneal rigidity including corneal hysteresis and corneal resistance factor together with the changes of Pentacam parameters could extend our knowledge on corneal physiology and could help to understand the underlying processes. Nevertheless, some authors failed to find a significant change in CCT over a decade^13,18^. It is to be mentioned that central and minimum corneal thickness values are clinically relevant. The former has an impact on the precise intraocular pressure value mainly in adults, while minimum corneal thickness has an importance during the accurate evaluation of corneal ectatic disorders. Therefore, physiological age-dependent decrease of these parameters should be considered when evaluating the progression of corneal ectatic disorders. We must aim to reliably predict progression and monitor the effectiveness of corneal cross-linking treatment in corneal ectatic disorders taking normal age-related cornea changes into account.

We have found that both keratometric values (K1 and K2) on the anterior corneal surface decreased significantly during follow-up, while K max remained stable. Adjusted statistical analysis of our data revealed that anterior surface keratometric values decrease stronger in younger than in older subjects. On the other hand, higher K values proved to be more stable over time independent of age. In a middle-aged to older population sample in a longitudinal study, K max increased 0.38 Diopters (p < 0.001) in 5 years^32^. In contrast with these results, Orucoglu *et al*. reported a significant positive correlation between anterior K1 and age, without any correlation between K2 and age^24^. In another cross-sectional study, no significant correlations were revealed between anterior keratometry and age^10^. These controversial results are possibly due to differences in study design, sample size and in age of participants. In earlier studies, a significant negative correlation was reported between posterior K1 and age^24^ and a significant positive correlation was revealed between posterior K2 and age^10^. In our study, unadjusted analysis shows no significant changes in K1 and K2 values of the posterior surface. However, adjusted statistical modeling revealed that the rate of change of keratometric values of the posterior corneal surface in the flat axis (K1B) may vary with baseline value and age. Low-range keratometric values increase significantly with time, while those in the high range do the opposite, suggesting a tendency for values to progress away from distributional extremes.

It is important to investigate corneal astigmatism with highly repeatable and reproducible methods such as Scheimpflug imaging to provide reliable data on age-related changes of astigmatism in order to improve long-term outcomes after intraocular lens implantation and refractive surgery^33–35^. Previous reports on age-related changes in astigmatism were all cross-sectional studies^8–10,24^ and/or were performed with other devices^6,7,36^. Total refractive astigmatism has been shown to change from with-the-rule to against-the-rule with age^4,7,24,32,36^. According to Ho *et al*. and Nemeth *et al*. who both used Pentacam^8,10^ there is an age-related shift towards against-the-rule and with-the-rule astigmatism for the anterior and posterior corneal surfaces, respectively. To the best of our knowledge, the current study is the first longitudinal study in the literature examining age-related changes of keratometric astigmatism at both the anterior and posterior surfaces of the cornea. Moreover, we examined keratometry values in a population covering the widest age range reported so far. In a cross-sectional and a longitudinal study alike, the prevalence of astigmatism increased with age^6,7^ however, in other cross-sectional studies it was demonstrated that keratometric astigmatism decreases significantly at both the anterior and posterior surfaces of the cornea with age^9,24^. Our findings are similar to these, although we only found evidence for keratometric astigmatism at the posterior surface to decrease significantly (p = 0.02), while astigmatism remained stable at the anterior surface. Moreover, posterior astigmatism showed a baseline-dependent significant increase from lower and a significant decrease from higher initial values, with no evidence of heterogeneity across baseline age (Fig. 2). Our findings are in contrast with the conclusions of Németh *et al*. that the posterior surface of the cornea is much more stable with advancing age than the anterior surface, even though there are no differences between the two studies in ethnic composition or in methods used^10^. Over time, the mean flat axis angle of the posterior keratometry shifted towards horizontal from a vertical (p = 0.004) or oblique (p = 0.004) baseline, which is in line with earlier studies proving that anterior corneal topographic astigmatism drifted from with-the-rule to against-the-rule astigmatism in older subjects^4,6–8,24^.

Vector analysis allows for a complete description of astigmatism characteristics^11^. In the assessment of anterior surface power vectors, Jackson’s cross cylinder power vector component at 45° showed significant changes with advancing age (unadjusted p = 0.047), which were explained by a substantial increase in older subjects with low to mid-range initial J45 values; values in younger subjects and in those with mid to high-range baseline readings did not change with advancing age (Fig. 2). Our findings are consistent with previous results that in addition to the frontal astigmatism, the J0 vector also remains stable with age. Based on our study with almost four years of mean follow-up, the axis of anterior astigmatism did not change significantly. In summary, our observations support the idea and have clinical relevance, because age-related changes of the anterior and posterior corneal surfaces should be considered when planning a surgical procedure for astigmatism correction^10^. Still, further longitudinal studies are needed to explore the true mechanism and effects of this phenomenon.

Age-specific changes of indices obtained with Pentacam have not been reported so far. Although they only give auxiliary information about the cornea, it is noteworthy that the indices of surface variation (ISV) and vertical asymmetry (IVA) did not seem to change during follow-up, but those of height asymmetry (IHA) and height decentration (IHD) increased significantly. The changes of these indices occurred in less than four years, with no evidence for heterogeneity across their baseline values.

Limitations of this study include a modest sample size precluding robust conclusions and the fact that only European descent of low refractive error (<1.5 diopters) were included. We do not know at this time, whether the changes observed in our study are linear with age. Moreover, because patients with high refractive error were excluded, we cannot estimate the effect of refractive surgery on the outcomes over decades. Despite these limitations, it is important to emphasize that our results highlight the fact that K1F, K2F, K1B, AstigB, PachyMin, and CV decreased significantly, IHA, IHD, and J45 vector increased significantly, and the mean AxisB shifted significantly with age.

The strength of our longitudinal study is that it gives reliable, reproducible data obtained with a Pentacam HR device^33–35^. Moreover, the study group consisted of 14 to 67-year-old individuals providing a wide age range to estimate age-dependent changes associating to different baseline ages. We explored for the first time that age and baseline value of corneal parameters have an effect on age-dependent corneal changes. To the best of our knowledge, this is the first prospective longitudinal study with a long follow-up period that reveals the clinically relevant consequences of aging on multiple parameters of the healthy cornea measured with Pentacam. Notably, this is the first longitudinal analysis showing that corneal thickness and volume reduce with age dependent on baseline thickness and baseline age. In conclusion, the results of our study add to existing knowledge on age-dependent changes of the anterior and posterior corneal surfaces. We demonstrate for the first time in the literature that age and baseline value may predict the direction and size of keratometric changes. Further longitudinal Pentacam studies with larger samples including high refractive errors and longer follow-up periods are required to validate our findings, and to confirm the clinical importance of changes of various parameters in refractive surgery, intraocular lens implantation, and also in the progression of corneal ectatic disorders.
